# Emergency Physician Twitter Use in the COVID-19 Pandemic as a Potential Predictor of Impending Surge: Retrospective Observational Study

**DOI:** 10.2196/28615

**Published:** 2021-07-14

**Authors:** Colton Margus, Natasha Brown, Attila J Hertelendy, Michelle R Safferman, Alexander Hart, Gregory R Ciottone

**Affiliations:** 1 Division of Disaster Medicine Department of Emergency Medicine Beth Israel Deaconess Medical Center Boston, MA United States; 2 Department of Emergency Medicine Harvard Medical School Boston, MA United States; 3 Department of Information Systems and Business Analytics College of Business Florida International University Miami, FL United States; 4 Department of Emergency Medicine Icahn School of Medicine at Mount Sinai New York, NY United States; 5 Department of Emergency Medicine Mount Sinai Morningside-West New York, NY United States

**Keywords:** COVID-19 pandemic, emergency medicine, disaster medicine, crisis standards of care, latent Dirichlet allocation, topic modeling, Twitter, sentiment analysis, surge capacity, physician wellness, social media, internet, infodemiology, COVID-19

## Abstract

**Background:**

The early conversations on social media by emergency physicians offer a window into the ongoing response to the COVID-19 pandemic.

**Objective:**

This retrospective observational study of emergency physician Twitter use details how the health care crisis has influenced emergency physician discourse online and how this discourse may have use as a harbinger of ensuing surge.

**Methods:**

Followers of the three main emergency physician professional organizations were identified using Twitter’s application programming interface. They and their followers were included in the study if they identified explicitly as US-based emergency physicians. Statuses, or tweets, were obtained between January 4, 2020, when the new disease was first reported, and December 14, 2020, when vaccination first began. Original tweets underwent sentiment analysis using the previously validated Valence Aware Dictionary and Sentiment Reasoner (VADER) tool as well as topic modeling using latent Dirichlet allocation unsupervised machine learning. Sentiment and topic trends were then correlated with daily change in new COVID-19 cases and inpatient bed utilization.

**Results:**

A total of 3463 emergency physicians produced 334,747 unique English-language tweets during the study period. Out of 3463 participants, 910 (26.3%) stated that they were in training, and 466 of 902 (51.7%) participants who provided their gender identified as men. Overall tweet volume went from a pre-March 2020 mean of 481.9 (SD 72.7) daily tweets to a mean of 1065.5 (SD 257.3) daily tweets thereafter. Parameter and topic number tuning led to 20 tweet topics, with a topic coherence of 0.49. Except for a week in June and 4 days in November, discourse was dominated by the health care system (45,570/334,747, 13.6%). Discussion of pandemic response, epidemiology, and clinical care were jointly found to moderately correlate with COVID-19 hospital bed utilization (Pearson *r*=0.41), as was the occurrence of “covid,” “coronavirus,” or “pandemic” in tweet texts (*r*=0.47). Momentum in COVID-19 tweets, as demonstrated by a sustained crossing of 7- and 28-day moving averages, was found to have occurred on an average of 45.0 (SD 12.7) days before peak COVID-19 hospital bed utilization across the country and in the four most contributory states.

**Conclusions:**

COVID-19 Twitter discussion among emergency physicians correlates with and may precede the rising of hospital burden. This study, therefore, begins to depict the extent to which the ongoing pandemic has affected the field of emergency medicine discourse online and suggests a potential avenue for understanding predictors of surge.

## Introduction

The contagiousness, fatality rate, and long-term sequelae thus far attributed to COVID-19, the disease caused by SARS-CoV-2, have led to significant strains on the health care system. Since the World Health Organization (WHO) first reported “a cluster of pneumonia cases” in Wuhan, China, on January 4, 2020 [1], the social media platform Twitter has become a source of both official health information and unofficial medical discourse regarding the ongoing pandemic. Boasting 180 million daily active users [2], not only does the service allow account holders to share links, media, and brief strings of text, but it has evolved into a public forum for unvetted information that can augment, if not supersede, more traditional dissemination methods.

On December 11, 2020, the US Food and Drug Administration (FDA) used Twitter to announce its authorization for immediate emergency use of the COVID-19 vaccine developed by Pfizer and BioNTech [3]. In its Twitter message, or *tweet*, about the decision, the FDA (@US_FDA) reiterated its aim to “assure the public and medical community that it has conducted a thorough evaluation of the available safety, effectiveness, and manufacturing quality information” [4]. Directly addressing Twitter’s medical community in this way was intentional: throughout the COVID-19 pandemic, many physicians turned to social media rather than traditional medical information channels to discuss the merits and demerits of possible treatments, prior to the availability of formal clinical guidance. Myriad treatment modalities and prevention strategies have been proposed at all levels, and Twitter has served as a means of disseminating everything from guidelines and data to anecdotes and opinions [5].

Utilizing social media to aid in the mapping of an ongoing crisis is not new, and Twitter use has previously been linked to, among others, the H1N1 and Zika virus epidemics [6,7]. Yet even with unparalleled international effort, formal forecasting models of COVID-19 have largely failed [8], and many geopolitical comparisons in popular media now in hindsight appear to have been premature [9-12]. As the front and, for many Americans, only door into the US health care system, emergency departments continue to be looked to for public health surveillance and treatment strategies, as a kind of finger on the epidemiological pulse of their communities [13,14].

Emergency physicians, in particular, have long been at the forefront of physician engagement with social media, relying on a budding network of fellow clinicians collaborating on what has become known as free open-access medical education [15]. The COVID-19 pandemic only further accentuates the unique role of the emergency physician community online, as frontline providers who not only take on substantial risk but who may also be able to provide substantial insight. Facing changed admission criteria, expanded alternate care sites, and recycled equipment, emergency physicians have been forced into the unenviable position of making difficult triaging and resource allocation decisions. This study, therefore, seeks to characterize the sentiment and topic trends in emergency physician discourse on Twitter throughout the prevaccination pandemic, as a potential harbinger of the surge needs that followed.

## Methods

### Sampling and Data Collection

This work was approved as exempt human subjects research through the Beth Israel Deaconess Medical Center Institutional Review Board in Boston, Massachusetts. In order to access Twitter’s application programming interface (API), a developer account was applied for and obtained. Python 3.8.5 and the Tweepy library (Python Software Foundation) [16] then made it possible to acquire all unique followers of the three major physician professional societies in emergency medicine: the American College of Emergency Physicians (ACEP; @ACEPNow), the Society for Academic Emergency Medicine (SAEM; @SAEMonline), and the American Academy of Emergency Medicine (AAEM; @aaeminfo) [17-21]. Because sex and gender are not directly recorded by Twitter but have previously been shown to influence social media engagement and even clinical diagnosis and management [22-24], gendered nouns and pronouns stated in user bios were considered in their place. Those users with privacy settings that would render tweets protected from analysis were removed. Each user bio was then initially screened by textual search for including any of the 157 text strings decided by the research team as connoting a public acknowledgement of one’s role as an emergency physician, such as “emergency medicine physician,” “emergency D.O.,” or “ER doc.”

Exclusion criteria included aspiring emergency physicians and students, organizations, physicians from other specialties, as well as users belonging to other professions, living outside the United States, or without a clear location at the state level. However, emergency physicians still in training, whether described as interns or residents, were not excluded. Exclusion for any of these reasons was determined by two practicing emergency physicians each reviewing and sorting all users manually and independently, with any discrepancies decided by consensus.

A chain-referral sampling method was then employed in order to expand the study group to include those US-based emergency physicians on Twitter not following one of the major professional organization accounts [25]. Followers of already-included participants were then aggregated to create a composite list of potential additional participants. After applying the same exclusions, this new group of users was then appended to the original as a more comprehensive sampling of US emergency physicians on Twitter.

All available tweets up to Twitter’s own limit of 3200 per user were acquired for each study participant. Tweets were removed if reposted as a *retweet* from a different post, if non-English, or if falling outside the study period from and including January 4, 2020, based on the date of the initial WHO announcement, to and including December 14, 2020, based on the date of the first FDA-approved vaccination in the United States [26].

### Sentiment and Topic Generation

Several different methods have previously been employed to conduct sentiment analysis of tweets specific to the health care field, with 46% of such tweets demonstrating sentiment of some kind [27]. Here, the open-source Valence Aware Diction and Sentiment Reasoner (VADER) analysis tool was used to determine both the direction and extent of tweet sentiment polarity, based on a lexicon of sentiment-related words. VADER has been shown to outperform human raters and, in handling emoji and slang, is particularly suited for social media text [28,29]. Sentiment polarity ratings were summed and standardized as a compound score between –1 and 1. By convention, tweets with a compound score between –0.05 and 0.05 were classified as neutral [29,30].

Using the gensim Python package, all tweets were tokenized and preprocessed, including removal of punctuation, special characters, mentions of other users, *stop words* of little topic value, and links to external websites. Hashtags, which users sometimes use to denote a contextual theme [31,32], were converted to text. Because frequently co-occurring words can exist with unique meaning, two- or three-word phrases were also considered as independent tokens, as in “healthcare_workers.” These preprocessed tweets then underwent unsupervised topic modeling in order to discern meaningful content themes. Latent Dirichlet allocation (LDA) is a common method for topic modeling that has previously been utilized to analyze health care–related tweets [33,34]. Topic coherence, as proposed by Röder et al due to its higher correlation with human topic ranking [35], was then maximized by iteratively modeling over a range of topic numbers as well as α parameters. The resulting topic model was then used to assign a dominant topic to each tweet included in the sample.

In order to contextualize these sentiment and topic trends within the ongoing pandemic, daily COVID-19 case counts and COVID-19 inpatient bed utilization (CIBU) rates were acquired from the US Centers for Disease Control and Prevention and the US Department of Health and Human Services [36,37]. These data were converted to 7-day simple moving averages to account for lower weekend reporting and other daily fluctuations [38,39]. Tweet volume, sentiment, and dominant topic trends were then correlated with new COVID-19 cases and CIBU through Prism, version 9.0.2 (GraphPad Software).

Further comparison between public health and Twitter data was made possible by plotting the 7- and 28-day simple moving averages and observing their intersection as a potential indicator for momentum using Excel, version 16.47.1 (Microsoft). Similarly, a moving average convergence/divergence oscillator (MACD) was generated by subtracting the 28-day exponential moving average from the 7-day exponential moving average. This MACD was then monitored for both (1) turning positive and (2) crossing above its own 7-day exponential moving average. These cross signals based on simple moving averages and on the MACD are both loosely derived from lagging indicators of historical price patterns that are commonly used in finance to guide investment decisions and have previously been applied directly to SARS-CoV-2 infection data [40,41].

## Results

**Figure 1:**
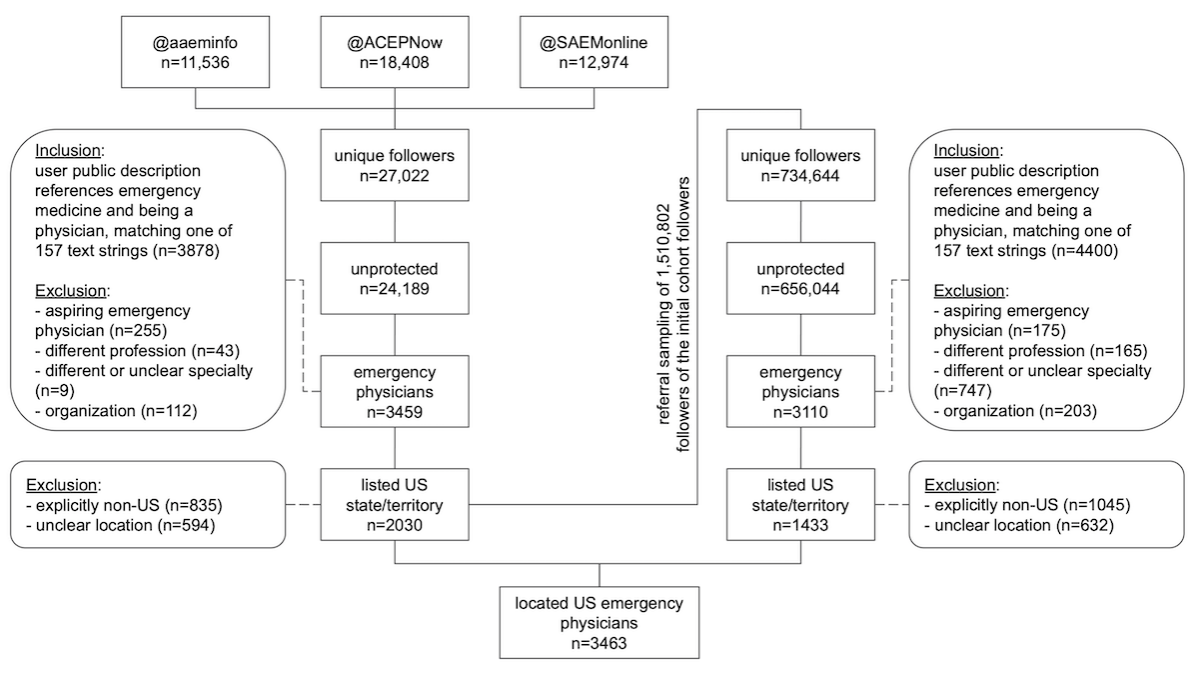
Overview of the methodology applied for study participant selection. Unprotected unique followers of the Twitter handles for three key US emergency physician professional organizations were sampled; they were included if referencing being an emergency medicine physician and excluded if not found to be an individual emergency physician located in a US state or territory. A referral sample of the original sample's followers underwent the same inclusion and exclusion criteria to contribute additional US-based emergency physicians to the study group. AAEM: American Academy of Emergency Medicine; ACEP: American College of Emergency Physicians; SAEM: Society for Academic Emergency Medicine.

The three key US emergency physician organizations had a collective 42,918 followers of their primary Twitter accounts as of December 11, 2020. When those following more than one professional organization were only counted once, there were 27,022 unique followers, with 10,905 (40.4%) belonging to at least two of the three groups (Figure 1). As an approximation for cohesion, the overlap coefficient of the three handles was 0.43, calculated as the ratio of the intersection over the maximum possible intersection ([A∩B∩C]/min[|A|,|B|,|C|]) [42]. After exclusions, 2073 US-based emergency physicians were identified, with high interrater reliability (κ=0.96).

There were 1,510,802 followers of the initial cohort acquired on December 12 and 13, 2020, with 734,644 found to be internally unique as well as distinct from the original user list assessed. Applying the same inclusion and exclusion criteria resulted in 3110 emergency physicians, 1433 of whom could clearly be identified as located in specific US states, territories, or districts through their public Twitter location and description (κ=0.94). Combining the two groups, there were 3463 US-based emergency physicians included in the study.

Study participants had been using Twitter for an average of 6.6 (SD 3.5) years, with an average of 183.8 (SD 491.0) total tweets (Table 1). Only 910 out of 3463 (26.3%) participants explicitly described themselves as a resident or intern currently in training. The most common US states represented were New York (433/3463, 12.5%) and California (395/3463, 11.4%), and the most contributory US region was the Northeast (1057/3463, 30.5%). Self-identified gender was infrequent (902/3463, 26.0%), with 466 of those 902 participants (51.7%) identifying as a man.

**Table 1:** Descriptive statistics of included US-based emergency physicians on Twitter.

Characteristic		Value (N=3463)
**Gender, n (%)**		
	Identified	902 (26.0)
	Men (n=902)	466 (51.7)
	Women (n=902)	436 (48.3)
	Unidentified	2561 (74.0)
**Usage**		
	Verified account, n (%)	27 (0.8)
	Duration (years), mean (SD)	6.6 (3.5)
	Tweets, mean (SD)	183.8 (491.0)
	Followers, mean (SD)	664.6 (5326.3)
	Since 2007-2009, n (%)	519 (15.0)
	Since 2010-2014, n (%)	1471 (42.5)
	Since 2015-2019, n (%)	1235 (35.7)
	Since 2020, n (%)	238 (6.9)
**Organizations followed, n (%)**		
	American Academy of Emergency Medicine (AAEM) only	144 (4.2)
	American College of Emergency Physicians (ACEP) only	351 (10.1)
	Society of Academic Emergency Medicine (SAEM) only	275 (7.9)
	AAEM and ACEP	114 (3.3)
	AAEM and SAEM	148 (4.3)
	ACEP and SAEM	343 (9.9)
	All three organizations	655 (18.9)
	None	1433 (41.4)
Training: identified as in training, n (%)		910 (26.3)
**US region, n (%)**		
	Midwest	789 (22.8)
	Northeast	1057 (30.5)
	South	884 (25.5)
	West	724 (20.9)
	Territory	9 (0.3)
**Top five US states, n (%)**		
	New York	433 (12.5)
	California	395 (11.4)
	Pennsylvania	249 (7.2)
	Texas	235 (6.8)
	Illinois	212 (6.1)

**Figure 2:**
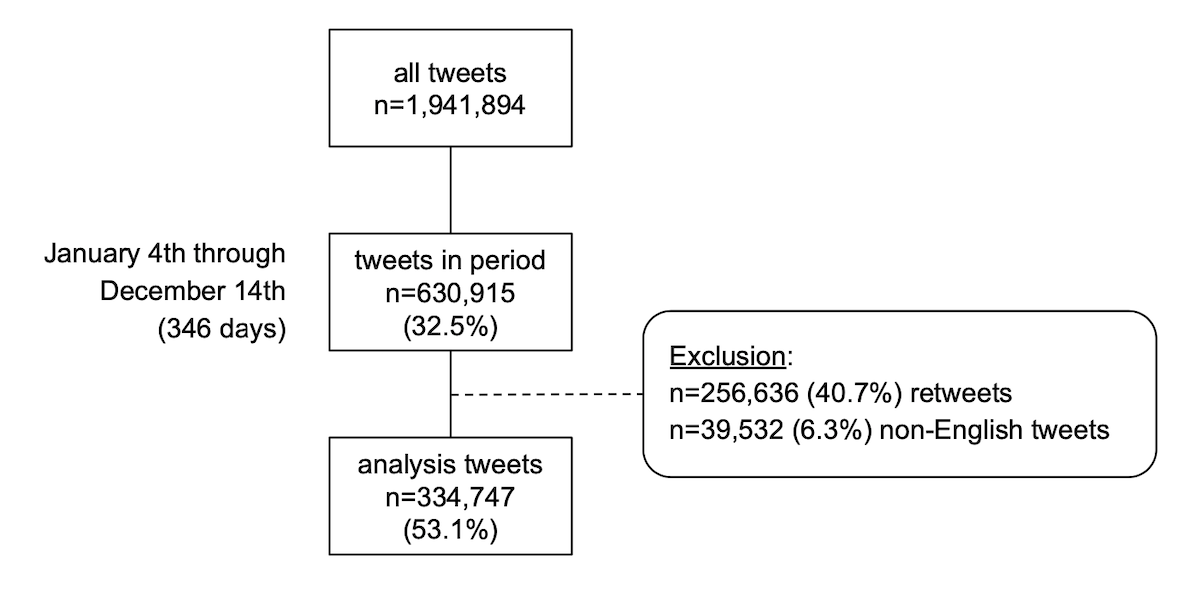
Overview of the methodology applied for tweets selected for analysis. Tweets collected for study participants were included if they fell within the January 4 through December 14, 2020, study period and excluded if they were found to be retweets, non-English tweets, and tweets of indeterminate language.

Tweets collected for the study group totaled 1,941,894 as of December 24, 2020, with 630,915 (32.5%) of those obtained falling between January 4 and December 14, inclusive (Figure 2). Because of a cap on the number of tweets able to be pulled through the official Twitter API, 44 out of 3463 (1.3%) users appeared to have exceeded the limit, such that not all tweets would have been captured. Despite truncation, these avid users still contributed 140,938 of all 1,941,894 (7.3%) collected tweets. Overall, 256,636 (40.7%) retweets and 39,532 (6.3%) non-English tweets were removed, leaving 334,747 (53.1%) unique English-language tweets for analysis. Daily volume went from a pre-March mean of 481.9 (SD 72.7) tweets to a mean of 1065.5 (SD 257.3) tweets thereafter.

After preprocessing, 1,958,230 semantic units, or *tokens*, were found within the corpus of tweets, with a total vocabulary of 12,401. LDA modeling over a range of topic numbers and parameters settled on a total of 20 content topics for this study. Two physicians then worked together to manually and jointly label these 20 topics based on discussion of key terms and representative tweets. For example, the topic with the top five terms of “resident,” “student,” “residency,” “learn,” and “year” was labeled as *medical training*. In this way, the most prevalent topics were found to relate to the *health care system* (45,570/334,747, 13.6%), *collaboration* (20,112/334,747, 6.0%), and *politics* (18,186/334,747, 5.4%) (Table 2). Notably, the *health care system* was the dominant topic throughout the study period, with two exceptions: it was supplanted from June 2 to 9 by *race relations* and from November 6 to 10 by *politics*.

**Figure 3:**
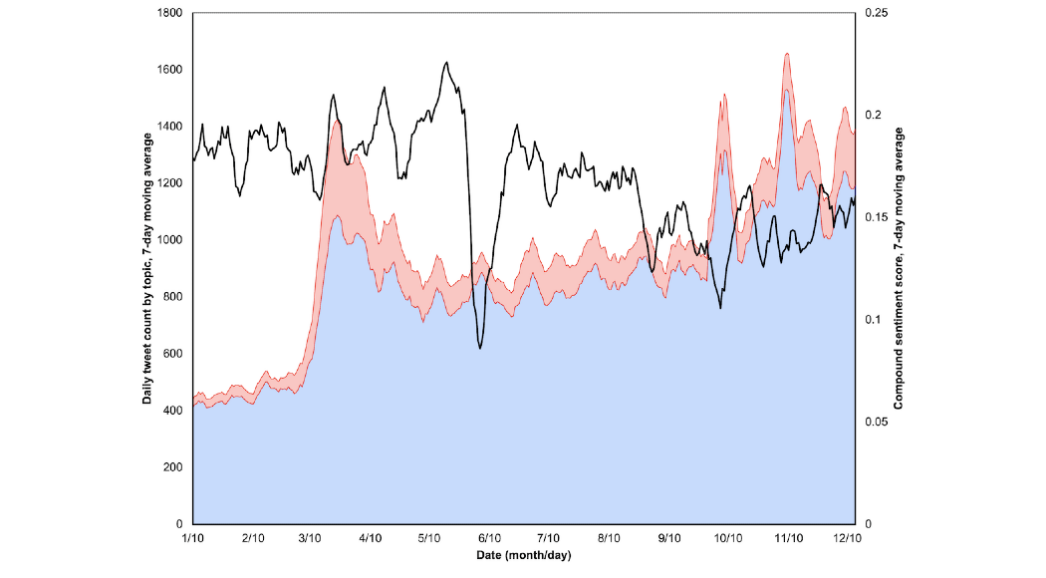
Stacked area plot of 7-day moving average daily counts of latent Dirichlet allocation–derived topics, both those pertaining to COVID-19 (red area) and those not (blue area) (left axis), plotted against the 7-day moving average of daily compound sentiment scores nationally (right axis).

Daily change in 7-day moving averages for specific tweet topics and sentiment polarity demonstrated small Pearson correlation coefficients for the topics of pandemic response (*r*=0.26, 95% CI 0.15-0.36), epidemiology (*r*=0.25, 95% CI 0.14-0.35), and clinical care (*r*=0.23, 95% CI 0.13-0.33) with reported COVID-19 cases (all *P*<.001) (Table 2). There was greater correlation for these topics with hospital bed utilization (all *P*<.001). While the three topics considered jointly were even more correlated (*r*=0.41, 95% CI 0.31-0.50), they still fell short of the correlation seen with the 9.4% of included tweets containing “covid,” “coronavirus,” “corona virus,” “cov-2,” “cov2,” or “pandemic” within the tweet text (*r*=0.47, 95% CI 0.38-0.56) (all *P*<.001). A proportional stacked area chart reveals an early overall increase in Twitter use as daily case counts rose, particularly among COVID-19–related topics (Figure 3). Aggregated sentiment scores reached a nadir on June 6, when *race relations* was the dominant topic in the sample, and again on October 6, the day after then–US President Donald Trump was discharged from his COVID-19 hospital admission [43].

**Table 2:** Topic descriptive statistics.

Topic label	Total tweets (N=334,747), n (%)	Compound sentiment score, mean (SD)	Case Pearson correlation, *r* (95% CI)	CIBU^a^ Pearson correlation, *r* (95% CI)	CIBU Spearman correlation, *r* (95% CI)	Key terms	Example tweet
Health care system	45,570 (13.6)	0.16 (0.36)	0.03 (–0.07 to 0.14)	0.12 (0.00 to 0.23)	0.07 (–0.05 to 0.18)	Care, health, physician, medicine, practice, system, medical, important, community, change, issue, lead, work, support, research, address, focus, policy, create, improve	“I’m not the one to ask about nursing. Nursing has always defined itself. The problem is the definition used to define ‘advanced nursing’ is the same definition used to define medicine. That is not the same definition that was used years ago, it changed. Common sense dictates one”
Collaboration	20,112 (6.0)	0.58 (0.34)	0.01 (–0.10 to 0.12)	0.09 (–0.03 to 0.20)	0.11 (–0.01 to 0.23)	Work, great, amazing, team, love, proud, congrat, congratulation, colleague, job, awesome, friend, support, part, good, hard, share, incredible, today, honor	“Honored to receive this award from @TXChildrensPEM^b^ section. Thank you all for being such a great group of mentors, colleagues, and friends! Also, winning the Fellow’s Award means so much. Happy for such a great group of fellows and mentees!”
Pandemic care	13,240 (4.0)	0.14 (0.50)	0.23 (0.13 to 0.33)	0.26 (0.15 to 0.37)	0.18 (0.06 to 0.29)	Patient, care, hospital, covid, doctor, nurse, emergency, doc, physician, call, sick, staff, visit, admit, treat, ed^c^, icu^d^, medical, work, room	“Physician-owned hospitals can increase the number of licensed beds, operating rooms, and procedure rooms by converting observation beds to inpatient beds, among other means, to accommodate patient surge”
Research	16,415 (4.9)	0.02 (0.47)	0.07 (–0.04 to 0.17)	0.06 (–0.06 to 0.17)	0.07 (–0.05 to 0.19)	Patient, study, treatment, high, give, low, risk, drug, pain, show, dose, trial, present, treat, disease, early, diagnosis, benefit, med, effect	“Take-homes from 2020 ACEP^e^ Opioids Clinical Policy: 1. Treat opioid withdrawal with buprenorphine. 2. Preferentially prescribe non-opioids for acute pain. 3. Avoid prescribing opioids for chronic pain. 4. Do not prescribe sedatives to patients taking opioids”
Race relations	15,128 (4.5)	–0.17 (0.51)	0.00 (–0.10 to 0.12)	0.03 (–0.09 to 0.14)	–0.02 (–0.14 to 0.09)	People, black, man, kill, woman, call, speak, matter, police, stand, white, stop, racism, word, racist, history, protest, happen, die, wrong	“Black lives matter means Black​ queer​ lives matter, Black ​trans ​lives matter, Black ​non-binary lives matter, Black ​femme​ lives matter, Black ​incarcerated ​lives matter, and Black ​disabled ​lives matter...”
Pandemic response	14,143 (4.2)	0.05 (0.50)	0.26 (0.15 to 0.36)	0.38 (0.28 to 0.47)	0.27 (0.16 to 0.38)	Covid, pandemic, coronavirus, vaccine, response, protect, health, virus, fight, ppe^f^, crisis, die, continue, country, worker, spread, leadership, expert, action, state	“#COVID. COVID COVID COVID COVID COVID COVID COVID COVID COVID 183,000+ Americans dead, and counting... Care for your neighbors. #WearAMask”
Reading	17,897 (5.3)	0.27 (0.41)	0.06 (–0.05 to 0.17)	0.20 (0.09 to 0.31)	0.13 (0.01 to 0.25)	Read, great, check, write, thread, article, post, book, list, follow, find, share, good, send, add, paper, twitter, email, tweet, link	“Please read the first paragraph of the new image again. It literally is saying what I originally replied with. Google searches do no good if you won’t read the text of what you find, not just the header.”
Schedule	15,577 (4.7)	0.15 (0.41)	0.04 (–0.07 to 0.15)	0.10 (–0.02 to 0.21)	0.07 (–0.05 to 0.19)	Day, time, week, today, hour, start, work, shift, year, long, wait, month, back, night, spend, end, run, sleep, minute, morning	“The length of shifts of studies in this paper started at 13 hours. Time off during day hours not post-night is obviously not the same as working 13 hours and having a few hours off before bed.”
Public safety	11,594 (3.5)	0.19 (0.45)	0.05 (–0.06 to 0.16)	0.26 (0.15 to 0.36)	0.12 (0.00 to 0.24)	People, school, safe, open, home, work, close, place, stay, follow, mask, risk, live, order, family, plan, community, back, person, kid	“Every single store we went into on Michigan Ave required a mask. Our hotel requires a mask anywhere inside. Even Millenium Park requires a mask to enter and walk around outside. And on the streets plenty of people are masked outside. I think compliance is excellent”
Politics	18,186 (5.4)	–0.01 (0.49)	0.00 (–0.11 to 0.11)	–0.01 (–0.13 to 0.10)	–0.05 (–0.17 to 0.07)	Vote, trump, election, lie, country, state, people, lose, president, win, debate, biden, stop, count, call, support, political, campaign, american, fact	“Trump’s personal lawyer: Guilty. Trump's campaign manager: Guilty. Trump’s deputy campaign manager: Guilty. Trump’s National Security Advisor: Guilty. Trump’s political advisor: Guilty.”
Entertainment	18,100 (5.4)	0.17 (0.47)	0.03 (–0.07 to 0.14)	0.05 (–0.07 to 0.16)	0.09 (–0.03 to 0.20)	Watch, good, play, love, guy, game, thing, time, bad, video, pretty, show, give, favorite, big, real, season, fan, idea, listen	“I only watched pro sports and news for decades, never watching any of the popular TV shows; now I’ve actually started watching Downton Abbey instead. I guess Breaking Bad or GOT is next. I haven’t seen a single episode of either. Any other suggestions?”
Epidemiology	14,208 (4.2)	0.01 (0.48)	0.25 (0.14 to 0.35)	0.36 (0.25 to 0.45)	0.17 (0.05 to 0.28)	Covid, test, case, death, number, testing, people, high, positive, report, rate, day, virus, infection, risk, coronavirus, symptom, spread, increase, rise	“Q: what if I traveled to high risk area/ contact w known #COVID19 case) & HAVE symptoms? A: Isolate yourself. U meet testing criteria but do not HAVE to get tested. If u test negative for everything, please isolate yourself until symptoms resolve as for any contagious illness.”
Scientific inquiry	13,235 (4.0)	0.13 (0.46)	–0.04 (–0.15 to 0.07)	0.12 (0.00 to 0.23)	0.12 (0.01 to 0.24)	Question, agree, datum, point, answer, science, study, base, evidence, fact, show, understand, opinion, true, wrong, important, good, correct, information, clear	“Many, including @realDonaldTrump, have abandoned science, logic and common sense Don’t take medical advice from charlatans Listen to real experts Hydroxychloroquine data shows no benefit + significant potential harms”
Protective equipment	15,156 (4.5)	0.14 (0.42)	0.07 (–0.04 to 0.18)	0.15 (0.03 to 0.26)	0.14 (0.03 to 0.26)	Mask, wear, put, hand, face, line, eye, time, head, find, leave, back, hold, room, pull, run, clean, cover, hair, remove	“A woman on the subway just pulled her mask down to blow her nose. Feeling like somehow people still don't get it...”
Business of medicine	12,217 (3.6)	0.12 (0.48)	0.07 (–0.04 to 0.18)	0.06 (–0.06 to 0.17)	0.12 (0.00 to 0.24)	Pay, money, system, physician, cost, make, free, health, state, give, problem, care, medical, job, insurance, company, hospital, healthcare, cut, plan	“Benchmarking to INW^g^ rates or lower, based on a antiquated federal fee scheduling system, is a non-starter for most physician owned and operated practices. Incentivize competition in the marketplace. Offer better reimbursement rates than CMGs^h^ or large groups. Break monopolies.”
Family	14,296 (4.3)	0.20 (0.46)	0.12 (0.01 to 0.23)	0.12 (0.00 to 0.23)	0.15 (0.03 to 0.26)	Year, kid, child, friend, family, good, call, give, time, talk, feel, parent, young, today, make, back, mom, remember, wife, baby	“Same with my wife and her parents back in the day.younger sister got everything she wanted. We married young and never asked for anything. Only her mother came to our wedding (teen marriage never lasts) 44 years ago...no wedding gifts.”
Lifestyle	17,610 (5.3)	0.18 (0.42)	–0.02 (–0.13 to 0.09)	0.07 (–0.05 to 0.18)	0.07 (–0.05 to 0.19)	Make, eat, food, car, good, run, water, dog, drive, walk, buy, love, drink, bring, hot, coffee, nice, thing, cool, enjoy	“Stuffed peppers: Cut 4 bell peppers in half lengthwise. In a skillet saute 2 cups spinach, 1/3 white onion and garlic. Add 1lb ground chicken. Season to taste. Add 1 cup cauliflower rice. Stuff the ‘rice’ into the peppers. Top peppers w/ cheese & bake for 20mins on 375 degrees.”
Medical training	15,994 (4.8)	0.36 (0.42)	0.08 (–0.03 to 0.19)	0.13 (0.01 to 0.24)	–0.03 (–0.15 to 0.09)	Resident, student, residency, learn, year, program, medical, medtwitter, great, join, today, virtual, mede, attend, interview, teach, school, talk, match, conference	“Thankful for my residency family today! Had a great week of shifts and an awesome virtual conference last week! My faculty and co-residents have been so amazing these last few months!”
Emotional reaction	12,401 (3.7)	0.14 (0.49)	–0.03 (–0.14 to 0.08)	0.10 (–0.01 to 0.22)	0.11 (–0.01 to 0.23)	Make, thing, good, feel, time, bad, people, hard, lot, happen, agree, change, easy, hear, decision, part, point, find, real, sense	“Are you nervous? Lots of people feel nervous when they come here That’s normal What are you nervous about? Are you nervous that something may hurt? A lot of people worry about that Nothing is going to hurt right now If that changes I’ll tell you & we’ll get thru it”
Inspirational	13,668 (4.1)	0.30 (0.50)	0.05 (–0.06 to 0.16)	0.14 (0.03 to 0.25)	0.17 (0.05 to 0.28)	Life, love, feel, hope, true, live, world, word, human, story, time, share, save, real, experience, moment, heart, family, find, change	“Thought of the day: I can share my earthly riches like peace, joy, time, talents, giftings, physical helps, hope, wisdom, emotional strength, encouragement, etc.”

^a^CIBU: COVID-19 inpatient bed utilization.

^b^TXChildrensPEM: Texas Children’s Hospital Pediatric Emergency Medicine.

^c^ed: emergency department.

^d^icu: intensive care unit.

^e^ACEP: American College of Emergency Physicians.

^f^ppe: personal protective equipment.

^g^INW: in-network.

^h^CMG: contract management group.

**Figure 4:**
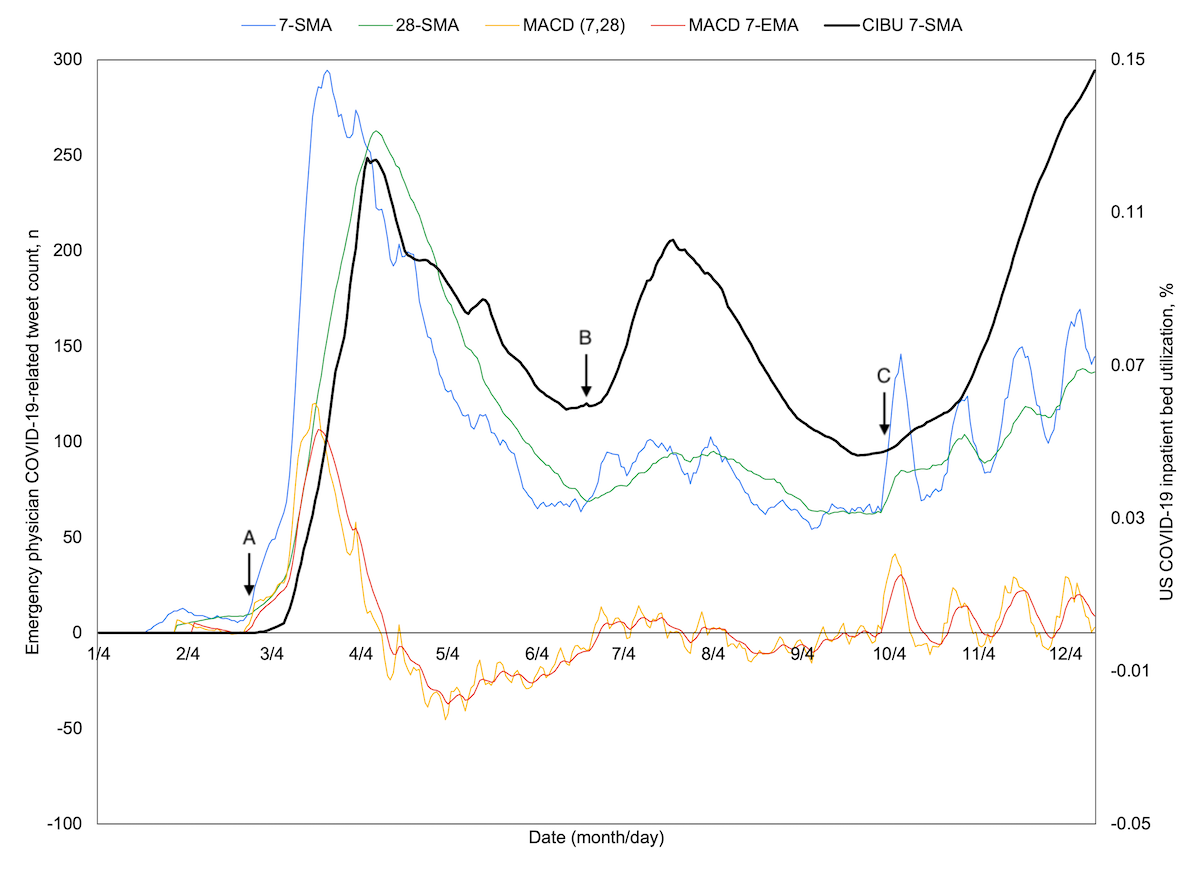
Time series plot of percent US COVID-19 inpatient bed utilization (CIBU; right axis) and its 7-day simple moving average (CIBU 7-SMA; right axis) against the 7-SMA and 28-day simple moving average (28-SMA) of COVID-19–related emergency physician tweets (left axis). Also plotted are the tweet exponential moving average convergence/divergence oscillator (MACD; left axis) and its own 7-day exponential moving average signal line (MACD 7-EMA; left axis). Labels A through C demonstrate sustained crossover points for tweet volume, where both the 7-SMA overcomes the 28-SMA and the MACD 7-EMA turns positive and overcomes the MACD as indicators of momentum.

Over the full study period, three peaks emerged in both COVID-19–related discussion and CIBU, with the rise in tweets appearing to precede the corresponding rise in CIBU. This may be better appreciated with attention directed to where the 7-day moving average crosses above the 28-day moving average as a signal of topic momentum (Figure 4). After the first recorded domestic COVID-19 case on January 22, 2020, February 25 was the first such cross and preceded a period of sustained increase in CIBU from February 28 to an April 9 peak. The next occurrence was on June 22, occurring alongside the second period of sustained increase in CIBU from June 15 to a peak on July 21. A brief cross on July 31 was short-lived, but the subsequent cross on September 13 was maintained and corresponded to a rise in COVID-19 hospital burden that started September 24 and continued through the remainder of the study period, with several episodic crosses seen thereafter. When the MACD is also considered, February 22 (Figure 4, point A) marks a cross above both the zero centerline as well as its 7-day moving average, 46 days before the April 9 peak in CIBU. The next such cross occurred on June 24 (Figure 4, point B), 27 days before the second peak, while the third surge in CIBU appeared to coincide with a first crossing on September 30 (Figure 4, point C).

**Figure 5:**
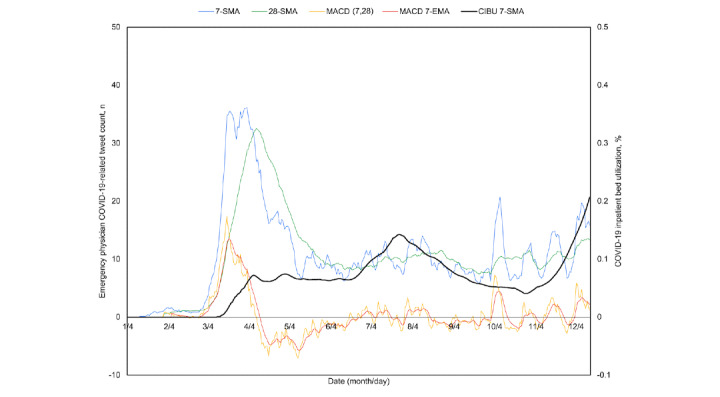
California time series plots of the 7-day simple moving average (7-SMA) in percent COVID-19 inpatient bed utilization (CIBU 7-SMA; right axis) against the 7-SMA and the 28-day simple moving average (28-SMA) of COVID-19–related emergency physician tweet count (left axis). Also plotted are the tweet exponential moving average convergence/divergence oscillator (MACD; left axis) and its own 7-day exponential moving average signal line (MACD 7-EMA; left axis).

**Figure 6:**
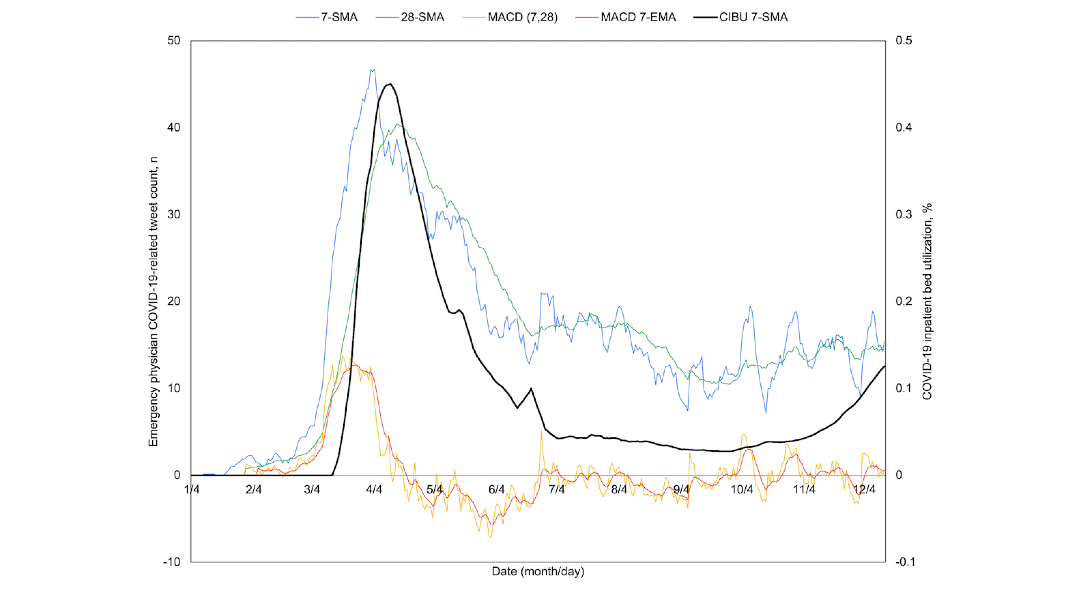
New York time series plots of the 7-day simple moving average (7-SMA) in percent COVID-19 inpatient bed utilization (CIBU 7-SMA; right axis) against the 7-SMA and the 28-day simple moving average (28-SMA) of COVID-19–related emergency physician tweet count (left axis). Also plotted are the tweet exponential moving average convergence/divergence oscillator (MACD; left axis) and its own 7-day exponential moving average signal line (MACD 7-EMA; left axis).

**Figure 7:**
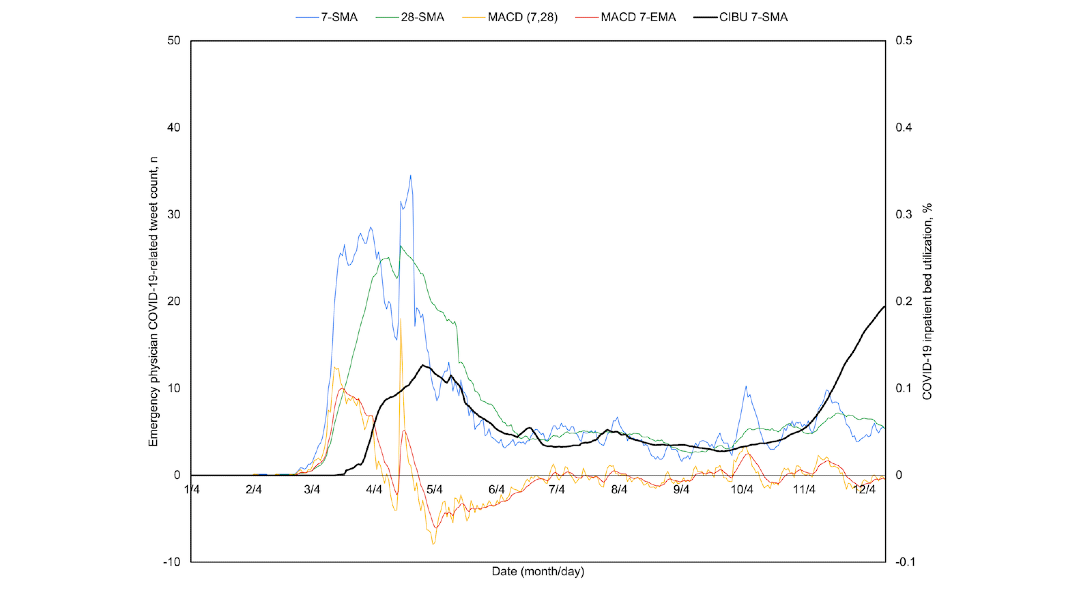
Pennsylvania time series plots of the 7-day simple moving average (7-SMA) in percent COVID-19 inpatient bed utilization (CIBU 7-SMA; right axis) against the 7-SMA and the 28-day simple moving average (28-SMA) of COVID-19–related emergency physician tweet count (left axis). Also plotted are the tweet exponential moving average convergence/divergence oscillator (MACD; left axis) and its own 7-day exponential moving average signal line (MACD 7-EMA; left axis).

**Figure 8:**
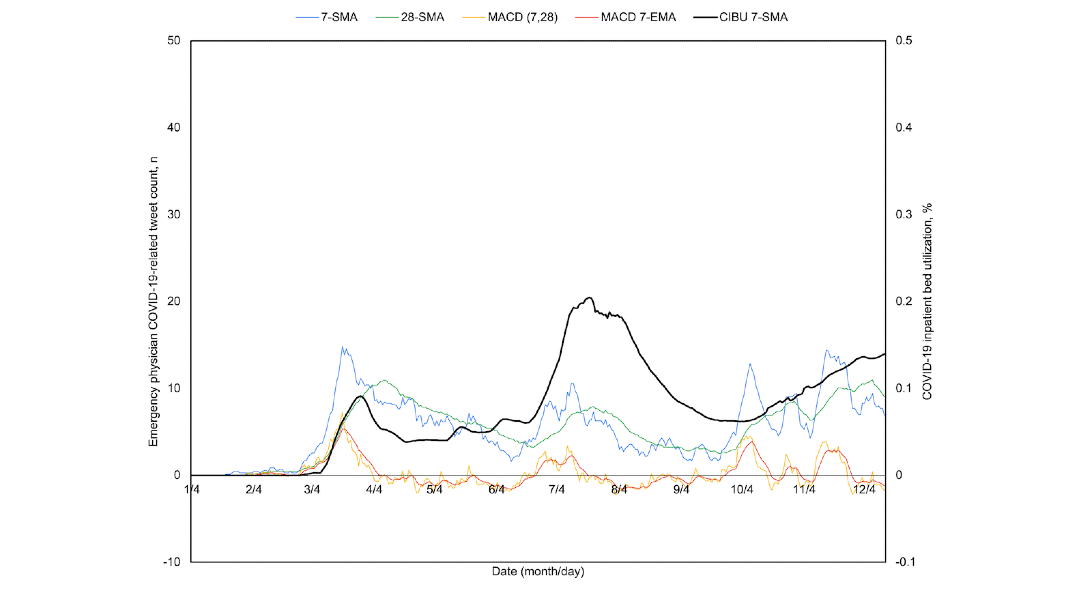
Texas time series plots of the 7-day simple moving average (7-SMA) in percent COVID-19 inpatient bed utilization (CIBU 7-SMA; right axis) against the 7-SMA and the 28-day simple moving average (28-SMA) of COVID-19–related emergency physician tweet count (left axis). Also plotted are the tweet exponential moving average convergence/divergence oscillator (MACD; left axis) and its own 7-day exponential moving average signal line (MACD 7-EMA; left axis).

Because the breadth and diversity of the United States may obfuscate local trends, the four most contributory states of California, New York, Pennsylvania, and Texas were similarly plotted (Figures 5-8). All four experienced a spring signal and subsequent surge, although New York has been recognized among them as an early epicenter [44]. Only Texas appears to have had a sustained cross of the 7-day moving average above the 28-day moving average from June 18 to July 15. This notably preceded the only significant summer peak among these states, reaching a maximum CIBU of 20.5% on July 20; in comparison, California reached a second peak of 14.3% on July 25 while neither New York nor Pennsylvania exceeded 10% again before November. The mean time from the preceding cross of moving averages in COVID-19–related emergency physician tweets to peak CIBU across the four states and the nation was 45.0 (SD 12.7) days.

## Discussion

### Principal Findings

Emergency physician engagement on Twitter has grown considerably since the start of the COVID-19 pandemic in both the topics raised and the sentiments conveyed. Furthermore, the analysis described here demonstrates conversations with increasing focus on pandemic response, clinical care, and epidemiology. That these topics correlate better with CIBU than simple case counts supports the idea that they may well serve as a kind of barometer of health care system strain. Momentum in these conversations, in fact, as shown by crossings of tweet count moving averages, were shown to occur before key rises in CIBU, which may with future research lend itself to the larger effort of predicting surge based on multiple data streams.

The COVID-19 pandemic has required emergency physicians to adapt continually to an extraordinary inadequacy of resources. While strains on the ventilator supply received much attention [45], early limitations in testing and bed availability also created significant clinical challenges [46], let alone the mental health effects that are likely to be far-reaching [47]. In the case of personal protective equipment (PPE) shortages, many frontline providers resorted to individual means to acquire makeshift supplies, and some even turned to social media, as in the case of the #GetMePPE Twitter hashtag, in order to spur necessary action [48-50]. Despite that potential good, such public distress and debate from the medical frontline has, at times, spurred controversy and even real-world, professional repercussions [51].

With overwhelming caseloads and without clinical consensus, frontline physicians have been forced to decide between various treatment modalities based on unclear and, at times, contradictory information, with significant moral distress [52]. The effort to maintain appropriate patient care despite these unknowns, when faced with a need for resource rationing [53], is a *de facto* implementation of crisis standards of care. While contingency planning is situational and incorporates some aspects of triage practiced routinely in overcrowded emergency departments across the country, the formal triggering of crisis standards, and implicit divergence from conventional standards, enacts systematic change in protocols and care plans during a sustained period of large-scale strain [54-56].

Taken together, this retrospective look at emergency physician Twitter use suggests a new way of considering the pandemic surge, as emergency physician utilization of Twitter reached unprecedented highs. There are likely several reasons for this. The online community has been shown to provide psychological benefit, potentially exacerbated by the isolation faced in providing crisis care, and by a perceived collapse in trust in the existing infrastructure and policy guidance [57,58]. That *collaboration* was the only topic among 20 to have a compound sentiment range that did not cross zero may relate to this yearning for support. Still, positive mean sentiment scores among the vast majority of topics were unexpected, given recent work pertaining to general public perceptions of the pandemic [59]. There may well be some sway to a self-perceived personal and professional connection to the dominant issue of the day. While the root cause is undoubtedly complex and multifaceted, the increase in emergency physician engagement with social media is likely here to stay.

Whether emergency physicians online can truly act as an early indicator for policy makers remains to be seen, but the community is undoubtedly a subset of the broader pandemic response and is worth looking at more closely. Moving averages have been used to indicate movement in financial markets but are not true predictors of future trends. Given the potential for false signaling and the challenge in determining what constitutes a sustained or meaningful cross prospectively, derived crosses of the kind shown here will likely need corroboration from a variety of other metrics as well as comparison to other samples and controls. Even so, the idea that an indicator of a physician behavioral trend online may also signal momentum in real-world hospital bed utilization has clear implications for the future. This is particularly relevant when it is considered that the study group itself was sourced directly from followers of major professional organizations for whom early recognition of surge would empower a more coordinated and efficacious policy response.

To make such a tool operationally relevant, collaboration between government and private sector partners will likely be necessary to build adequate data pathways allowing for public health surveillance in real time. While emergent topic generation from a retrospective corpus of tweets is not a feasible option for rapid and predictive modeling, this work suggests that even a simple collection of tweets containing disease-specific text strings can nonetheless yield important, potentially meaningful information to inform resource allocation and other policy decisions. Ultimately, all disasters are media events, affecting both how and what information is conveyed. There is, therefore, no great leap of faith in acknowledging that social media, too, may have an important part to play. Future research must delve further into how such tools can one day be used in the early recognition of, and response to, health care strain of such magnitude.

### Limitations

In holding to strict inclusion criteria, this study overlooked Twitter users who were not explicit in their self-identification as practicing emergency physicians. Emergency physicians were made the narrow focus of this work based on their key roles as clinical decision makers overseeing department throughput, but inclusion of nurses, technicians, and nonphysician midlevel providers may add breadth. Additionally, follower referral from within the sample may have introduced bias that could have been avoided by subsampling with some individuals selected for study participation and others only for referral [25]. Even so, a comprehensive list of emergency physicians on Twitter compiled in 2016 concluded that there were only 2234 such users around the globe [60]. Social media use has undoubtedly risen since, particularly with the influx of a growing number of emergency medicine residents [61], but the sample provided here does appear to be appropriately sized for this purpose.

Even so, this sample size was insufficient in both participant number and geographic spread to allow for more granular geographic analysis by city or county, although public health surveillance often occurs at this level [62,63]. Both demographic characteristics and spaciotemporal effects at the level of the individual participant have previously been shown to bias tweet sentiment and content, but these were not controlled for in this study [64,65].

Reliance on Twitter may itself limit generalizability, given its comparatively higher representation of young, urban, and minority users when compared with the general US population [66]. Only one social network platform was analyzed, and, insofar as it serves as a public forum, what medical professionals say online does not necessarily correlate with what they think or feel [67]. The study group, however, was not aware of its participation in this research, thereby avoiding that influence on behavior [68]. Excluding reposted tweets may have overlooked certain sentiments and reactions. Additionally, LDA topic modeling depends not only on the size of the overall corpus but on the length of the individual documents themselves. Although methods such as aggregation into larger documents have been proposed in order to overcome tweet brevity [69], doing so would not have allowed for the temporal and user-specific analysis intended. Finally, care must be taken in interpreting relationships between variables, such as physician tweets and CIBU, when both variables have undergone averaging or smoothing, which can sometimes suggest correlation where none exists.

### Conclusions

This work reveals both the opportunity and the pressing need to explore social media use by the emergency physician community as a means of anticipating surge needs. By acting as gatekeepers to the hospital, emergency physicians are uniquely positioned to act as early indicators of hospital surge, and finding methods such as Twitter usage, which can track and analyze these indicators, could be vital to future pandemic planning and response.

## Abbreviations

AAEM: American Academy of Emergency Medicine
ACEP: American College of Emergency Physicians
API: application programming interface
CIBU: COVID-19 inpatient bed utilization
FDA: Food and Drug Administration
LDA: latent Dirichlet allocation
MACD: moving average convergence/divergence oscillator
PPE: personal protective equipment
SAEM: Society for Academic Emergency Medicine
VADER: Valence Aware Diction and Sentiment Reasoner
WHO: World Health Organization
